# Sex, Pramipexole and Tiagabine Affect Behavioral and Hormonal Response to Traumatic Stress in a Mouse Model of PTSD

**DOI:** 10.3389/fphar.2021.691598

**Published:** 2021-06-30

**Authors:** Natalia Malikowska-Racia, Kinga Salat, Joanna Gdula-Argasinska, Piotr Popik

**Affiliations:** ^1^ Department of Behavioral Neuroscience and Drug Development, Maj Institute of Pharmacology, Polish Academy of Sciences, Krakow, Poland; ^2^ Department of Pharmacodynamics, Faculty of Pharmacy, Jagiellonian University Medical College, Krakow, Poland; ^3^ Department of Radioligands, Faculty of Pharmacy, Jagiellonian University Medical College, Krakow, Poland; ^4^ Faculty of Health Sciences, Jagiellonian University Medical College, Krakow, Poland

**Keywords:** Pramipexole, tiagabine, corticosterone, PTSD, trauma, sex, HPA

## Abstract

Posttraumatic stress disorder (PTSD) has been associated with abnormal regulation of the hypothalamic-pituitary-adrenal gland axis (HPA). Women demonstrate a more robust HPA response and are twice as likely to develop PTSD than men. The role of sex hormones in PTSD remains unclear. We investigated whether post-trauma chronic treatment with the GABA-ergic agent tiagabine and dopamine-mimetic pramipexole affected the behavioral outcome and plasma levels of corticosterone, testosterone, or 17β-estradiol in female and male mice. These medications were investigated due to their potential capacity to restore GABA-ergic and dopaminergic deficits in PTSD. Animals were exposed to a single prolonged stress procedure (mSPS). Following 13 days treatment with tiagabine (10 mg/kg) or pramipexole (1 mg/kg) once daily, the PTSD-like phenotype was examined in the fear conditioning paradigm. Plasma hormones were measured almost immediately following the conditioned fear assessment. We report that the exposure to mSPS equally enhanced conditioned fear in both sexes. However, while males demonstrated decreased plasma corticosterone, its increase was observed in females. Trauma elevated plasma testosterone in both sexes, but it had no significant effects on 17β-estradiol. Behavioral manifestation of trauma was reduced by pramipexole in both sexes and by tiagabine in females only. While neither compound affected corticosterone in stressed animals, testosterone levels were further enhanced by tiagabine in females. This study shows sex-dependent efficacy of tiagabine but not pramipexole in a mouse model of PTSD-like symptoms and a failure of steroid hormones’ levels to predict PTSD treatment efficacy.

## Introduction

Traumatic stress triggers a maladaptive response of the hypothalamus-pituitary-adrenal gland axis (HPA), evidenced as abnormally fast negative feedback (Yehuda et al., 1993) that is one of the core hallmarks of posttraumatic stress disorder (PTSD). However, the mechanism by which HPA adaptability contributes to PTSD has not yet been fully explained; for a review, see Dunlop and Wong (2019). The twice higher prevalence of PTSD in women (Kessler et al., 1995; Yehuda et al., 2015) coincides with sex-dependent HPA responsiveness (Heck and Handa, 2019). Considering that sex affects the response to stress and thus PTSD susceptibility, we hypothesized that it might also affect PTSD’s pharmacotherapy efficacy.

To address this issue in an animal model of PTSD, we used the mouse single prolonged stress (mSPS) procedure, which resembles human exposure to traumatic stress (Perrine et al., 2016). Trauma exposure predominantly affects fear conditioning, which manifests as an exaggerated fear reaction that tends to generalize, and fails to extinguish (Orr et al., 2000; Morey et al., 2015). The homology of conditioned fear between non-human mammals and humans is striking and constitutes face and construct validities of the mSPS and SPS models (VanElzakker et al., 2014; Perrine et al., 2016; Verbitsky et al., 2020).

Here we examined the effects of pramipexole, an antiparkinsonian D_2_-like receptor agonist, which reduced conditioned freezing and decreased immobility in male mice exposed to mSPS (Malikowska-Racia et al., 2019a). Pramipexole may improve trauma memory extinction (Gerlicher et al., 2018). Furthermore, as the D_3_-preferring agonist, pramipexole aims at the promising target for the treatment of neuropsychiatric disorders, which share some common symptoms with PTSD (Leggio et al., 2019; Leggio et al., 2021; for the review, see; Kiss et al., 2021). The concept of D_3_ receptors' role in PTSD has already been proposed, being further discussed in Torrisi et al. (2019). Nevertheless, the rationale of using pramipexole in this study primarily relies on the hypothesis that women and men trauma victims present different, dopamine-dependent adaptation to stress. Increased dopamine levels following stress have been shown to prevent abnormal HPA response and promote adaptive coping strategies; for the review, see Cabib and Puglisi-Allegra (2012). This mechanism is sex-dependent as following an uncontrollable stressor, female rats display prolonged passive coping and greater VTA dopamine decline than males (Rincón-Cortés and Grace, 2017). Thus, considering that dopamine-driven stress adaptation in females is not as effective as in males, we hypothesized that dopamine-mimetic medications, such as pramipexole, would demonstrate higher efficacy in traumatized female mice.

Another compound investigated here was the GABA-mimetic anticonvulsant medication tiagabine, which in our earlier report (Malikowska-Racia et al., 2019b) decreased immobility and anxiety following acute administration in mSPS-exposed male mice. Of note, GABA-ergic deficits have been demonstrated in PTSD patients (Geuze et al., 2008; Trousselard et al., 2016; Rasmusson et al., 2019), most likely resulting from an insufficient neurosteroid modulation of GABA-A receptors. Since impaired neurosteroidogenesis as found in PTSD females further attenuates GABA-ergic tone (Pineles et al., 2018; Rasmusson et al., 2019), we hypothesized that the efficacy of tiagabine, which inhibits GABA reuptake, would be greater in female mice. This is because the positive modulation of GABA-A receptors conferring neurosteroid sensitivity is fundamental for tonic HPA inhibition (Sarkar et al., 2011).

To closely model the clinical setting, we treated mice almost immediately after trauma. It has been shown that there is a “golden hours” time window in humans exposed to trauma, during which preventive treatment should be initiated (Carmi et al., 2016). Mice were treated with tiagabine or pramipexole for the following two weeks, i.e., up to the time they showed PTSD-like symptoms. Following sub-chronic treatment and behavioral tests, we examined the levels of plasma corticosterone as well as testosterone and 17β-estradiol because sex steroids have been shown to modify HPA response (Heck and Handa, 2019).

## Materials and Methods

### Chemical Compounds Used in the Study

Pramipexole dihydrochloride (1 mg/kg, s.c.; Sigma Aldrich, Poland) and tiagabine (10 mg/kg, i.p., Tocris Bioscience, Germany) were dissolved in 0.9% saline or suspended in 1% Tween 80, respectively. Both medications were injected in a volume of 10 ml/kg once daily at 8:00–9:00 AM for 13 days. Doses were selected based on our previous studies (Salat et al., 2015; Malikowska-Racia et al., 2019a). Control groups received 0.9% saline solution (10 ml/kg). Isoflurane, used for anesthesia in the mSPS procedure, was provided by Baxter (Puerto Rico, United States).

### Ethics, Animals, and Housing Conditions

All procedures were approved by the 1^st^ Local Ethics Committee of the Jagiellonian University in Kraków (292/2019) and were in full accordance with ethical standards laid down in respective Polish and EU regulations (Directive No. 86/609/EEC). Subjects were 48 male and 48 female albino Swiss CD-1 5-week old mice, weighing 18–22 g at the arrival to the laboratory. The animals were purchased from the Animal Breeding Farm of the Jagiellonian University Faculty of Pharmacy.

The animals were kept in groups of eight in standard plastic cages (42 × 26.5 × 18 cm) and were housed under controlled conditions (room temperature of 22 ± 2 °C, light/dark (12:12) cycle, lights on at 7:00 AM, humidity 50–60% and free access to food and water). For the tests, the animals were selected randomly. All experiments were performed between 8:00 AM and 3:00 PM in a sound-attenuated room under dim light and continuous white noise of 65 dB.

### Experimental Schedule

**FIGURE 1:**
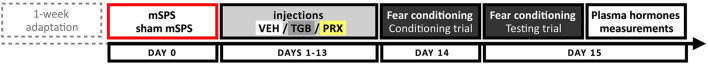
Experimental schedule. Male and female mice were exposed to mSPS (N = 24 + 24) or sham mSPS (N = 24 + 24). Twenty-four hours following trauma, 13-days long treatment with tiagabine (TGB, 10 mg/kg, i.p.) pramipexole (PRX, 1 mg/kg, s.c.) or saline began. On days 14–15, conditioned fear was examined, and 1 h later, the animals were decapitated, and blood samples were collected for further evaluation of plasma corticosterone, testosterone, and 17β-estradiol (for each hormone and sex, *N* = 8). Abbreviations: mSPS – mouse single prolonged stress, PRX – pramipexole, TGB – tiagabine.

Mice were exposed to mSPS or sham mSPS procedure at their age of 6 weeks. Twenty-four h following trauma, the treatment with pramipexole, tiagabine or saline began. Following 13 days administration, on days 14–15, the fear conditioning test was performed. Afterward, mice were decapitated, and blood samples were collected to determine corticosterone, testosterone, and 17β-estradiol plasma levels. The study timeline is presented in Figure 1.

### Behavioral Procedures

#### Mouse Single Prolonged Stress Procedure

Mouse mSPS was done as in Perrine et al. (2016) with some modifications (Malikowska-Racia et al., 2019a). Briefly, mice were exposed to a series of four different stressors: the tight restrainment (2 h), water forced group swim (10 min) and the exposure to a rat predator scent (15 min). Due to the current ethical requirements, the original diethyl ether anesthesia (Perrine et al., 2016) has been replaced with isoflurane anesthesia lasting for 3–4 min. Control mice underwent a sham-mSPS procedure, which consisted of handling and weighing. Next, all mice were left in the home cages for an undisturbed period of 24 h.

#### Contextual Fear Conditioning

Contextual fear conditioning was performed as reported (Malikowska-Racia et al., 2019a), except for an auditory cue's absence. We used a white-painted cage (Bioseb, France; 25 × 18 × 16 cm), floored with metal plates that delivered electroshocks. On the first day, each mouse was individually placed in the cage for a 1 min habituation period. During the next 2 min, each animal was exposed to five electric shocks (duration: 2 s, current intensity: 0.8 mA). The next day mice were placed individually in the same cage again, and after a 30 s habituation period, the 2 min testing period started. The dependent measure, i.e., the freezing response time defined as the total immobility excluding movements enabling respiration, was recorded and then scored manually by a trained observer blind to the treatment conditions.

### Measurement of Steroid Hormones

Animals were decapitated between 10:00 AM and 12:00 AM, and trunk blood was collected and centrifuged (2000 × g, at 4°C for 10 min) to collect plasma. Levels of corticosterone, testosterone, and 17β-estradiol were measured using commercially available ELISA kits (Neogen Corp., Lexington, United States) following the manufacturer’s instructions. The samples and the standards were run in duplicates. We used three types of 96-well plates (corticosterone #4028110; testosterone #402510, 17β-estradiol #402110). The horseradish peroxidase was added to the antibody-coated plate and incubated for 1 h at room temperature (∼24°C). Following incubation, the plate was washed three times with a wash buffer provided with the kit. Next, the mixture of tetramethylbenzidine and H_2_O_2_ was added and incubated for 30 min at room temperature. The bound conjugate was detected using a plate reader (POLARstar^®^ Omega, BMG LABTECH, Germany) at an absorbance of 650 nm. Data were quantified based on the standard calibration curves.

### Statistical Analysis

For statistical analysis, the IBM SPSS Statistics (v.24, NY, United States) was used.

Using 3-way ANOVA, we first investigated the effects of sex, treatment, and mSPS as well as whether sex affected treatment efficacy, whether sex affected mSPS effects, and whether treatment affected mSPS response. The sex x treatment x mSPS interaction was examined to determine whether treatment efficacy was similar in both sexes and depended on mSPS.

**FIGURE 2:**
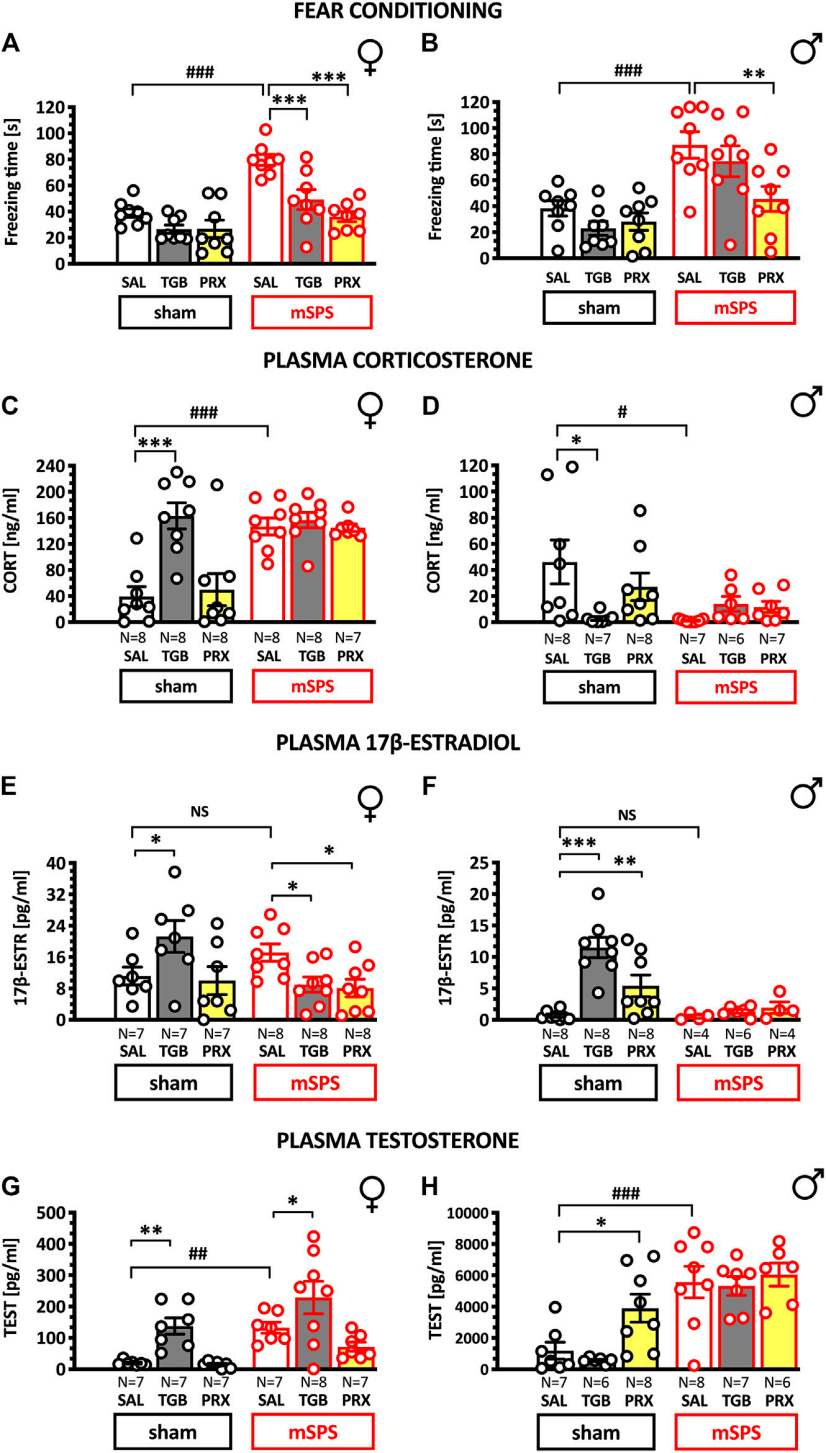
Effects of 13 days treatment with pramipexole and tiagabine on freezing duration in the fear conditioning test **(A,B)**; corticosterone **(C,D)**, 17β-estradiol **(E,F)** and testosterone **(G,H)** plasma levels in female and male mice subjected to PTSD-like set of stressors two weeks earlier. Mice were exposed to mSPS or sham mSPS, and 24 h later, the 13 days long treatment with saline (SAL), tiagabine (TGB; 10 mg/kg, i.p.) or pramipexole (PRX; 1 mg/kg, s.c.) has started. Drugs were given once daily at 8:00–9:00 AM. Following fear conditioning performed on days 14–15, animals were decapitated, and the blood samples were collected. Results are presented as means ± SEM. Data were analyzed using contrast analyses following 3- or 2-way ANOVA. Note different maximum ordinate values for female and male mice data. Symbols: *p < 0.05, **p < 0.01, ***p < 0.001 vs. saline groups tested in the same stress conditions; ^#^ *p* < 0.05, ^##^ *p* < 0.001 vs. same-treatment groups tested in sham mSPS conditions. Data below the quantification limit of hormonal assays were excluded from the analyses. Abbreviations: 17β-ESTR–17β-estradiol, CORT—corticosterone, mSPS–mouse single prolonged stress, PRX – pramipexole, SAL – saline, TEST – testosterone, TGB–tiagabine.

Since the sex x treatment x mSPS interaction was not significant for fear conditioning and estradiol levels’ data (Figures 2A,B, Figures 2E,F, respectively), and because testosterone levels were ∼ 20 x higher in males than females (Figures 2G,H), these data sets were then subjected to two independent 2-way ANOVAs for each sex. It served to examine whether mSPS and/or treatment affected the given phenotype and whether the effects of mSPS depended on the treatment. The two *a priori* questions: 1) whether mSPS affected the dependent variable in vehicle-controls and, 2) whether treatment with tiagabine or pramipexole exerted different effects than the respective vehicle controls, were assessed with contrast analyses (Cardinal and Aitken, 2006).

A linear regression model and correlation analyses were used to determine *r*, *R* ^*2*^ and statistical significance of the relationship between freezing time duration and plasma hormones levels.

The detailed statistics are presented in the Supplementary Material.

Sample sizes were estimated with the use of G*Power Software (v. 3.1.9.4.) (Perugini et al., 2018), which indicated minimal sample size *N* = 5 and actual power of 0.8 for ANOVAs (Cohen’s *f* = 0.45; α = 0.05) or *N* = 4 and actual power of 0.83 for contrast analyses (Cohen’s *d* = 2.5; α = 0.05).

## Results

### Effects of mSPS, Pramipexole and Tiagabine on Conditioned Fear

Since 3-way ANOVA showed no significant sex x treatment x mSPS interaction, data were analyzed with two 2-way ANOVAs. Both females (Figure 2A) and males (Figure 2B) exposed to mSPS presented prolonged freezing [mSPS effect for females: F(1,42) = 34.184 and males: F(1,42) = 31.309, *p* < 0.001]. Treatment affected freezing in both sexes [F(2,42) = 16.41, *p* < 0.001 and F(2,42) = 4.52, *p* < 0.05 for females and males, respectively], and treatment efficacy depended on stress exposure in females but not males [mSPS x treatment: F(2,42) = 4.787, *p* < 0.05 and F(2,42) = 2.418, NS, respectively].

Contrast analyses revealed that mSPS increased freezing both in females [*t*(42) = 5.656, *p* < 0.001] and males: [*t*(42) = 4.012, *p* < 0.001]. In stressed females both tiagabine [*t*(42) = 4.221] and pramipexole [*t*(42) = 6.048, *p* < 0.001] reduced conditioned freezing. In stressed males, only pramipexole reduced conditioned freezing [*t*(42) = 3.411, *p* < 0.01]. In the sham conditions, none of the medications affected the freezing response (Figures 2A,B).

These data suggest that mSPS applied two weeks earlier increased conditioned freezing and that pramipexole effectively reduced it in both sexes, while tiagabine reduced it in females only.

Effects of mSPS, pramipexole and tiagabine on plasma corticosterone level.

Figures 2C,D demonstrate that mSPS exposure [F(1,78) = 9.33, *p* < 0.01], sex [F(1,78) = 154, *p* < 0.001] and treatment [F(2,78) = 4.5, *p* < 0.05] affected plasma corticosterone as revealed by 3-way ANOVA. The sex x treatment x mSPS interaction was also significant [F(2,78) = 10.03, *p* < 0.001], suggesting complex inter-relation among these factors.

Sex modified the long-term effects of mSPS [F(1,78) = 25.96, *p* < 0.001] and contrast analyses revealed corticosterone increase in stressed females as compared to sham-mSPS females [*t*(78) = 5.672, *p* < 0.001; Figure 2C]. In males, trauma exerted an opposite effect in that a decrease of corticosterone levels was observed in stressed- [*t*(78) = 2.29, *p* < 0.05] as compared to sham-mSPS subjects; Figure 2D.

The treatment produced different effects on plasma corticosterone in males and females [treatment x sex F(2,78) = 10.34, *p* < 0.001]. Specifically, due to tiagabine, corticosterone levels were higher in non-stressed females [*t*(78) = 6.529, *p* < 0.001] and lower in non-stressed males [*t*(78) = 2.208, *p* < 0.05] as compared with respective saline controls. However, either tiagabine or pramipexole failed to affect corticosterone levels in traumatized mice.

These data suggest that mSPS increased and decreased corticosterone in females and males respectively. Neither tiagabine nor pramipexole affected corticosterone response in traumatized animals. In sham mice, however, tiagabine increased corticosterone in females and decreased it in males. The effects of tiagabine somewhat resemble the effects of trauma because mSPS *per se* also increased corticosterone in females and decreased it in males.

### Effects of mSPS, Pramipexole and Tiagabine on Plasma Estradiol Level

While 3-way ANOVA demonstrated significant effects of sex, mSPS and treatment on estradiol plasma levels, it also revealed insignificant mSPS x treatment x sex interaction. Thus, data were further analyzed with two separate 2-way ANOVAs in females and males. These analyses revealed significant treatment x stress interaction in females [F(2,39) = 5.539, *p* < 0.01 and males F(2,32) = 7.67, *p* < 0.01], suggesting that the treatment effect depended on prior stress exposure. Nonetheless, neither in females (Figure 2E) nor in males (Figure 2F), prior exposure to mSPS affected plasma estradiol levels, suggesting that this hormonal phenotype might not be associated with PTSD-like symptomatology.

Of note, in non-stressed females [*t*(39) = 2.513; *p* < 0.05] and males [*t*(32) = 6.724; *p* < 0.001] tiagabine increased plasma estradiol levels. In contrast, in mSPS exposed females but not males, both tiagabine [*t*(39) = 2.17; *p* < 0.05] and pramipexole [*t*(39) = 2.4; *p* < 0.05] reduced this hormone level. This may suggest that in females, reduced estradiol levels could be associated with the treatment efficacy. Tiagabine-induced estradiol increase in non-stressed females (Figure 2E) resembling a similar corticosterone increase (see Figure 2C).

Overall, these data suggest that plasma estradiol levels are not indicative of the prior severe stress in mice in the present laboratory conditions. Because tiagabine increased plasma estradiol levels in non-stressed mice of both sexes and decreased this hormone level in stressed females, one may conclude that the effects of tiagabine depended on prior stress exposure.

### Effects of mSPS, Pramipexole and Tiagabine on Plasma Testosterone Level

Because testosterone levels were, as expected, much higher in males, data were *a priori* analyzed with separate 2-way ANOVAs in males and females. These analyses revealed that treatment efficacy did not depend on prior stress exposure in females [F(2,37) = 0.749] and in males [F(2,36) = 1.681]. Nonetheless, contrast analyses revealed that in females [*t*(37) = 2.869; *p* < 0.01; Figure 2G] and in males [*t*(36) = 4.213; *p* < 0.001; Figure 2H] the prior exposure to mSPS increased testosterone levels.

Neither treatment with pramipexole nor tiagabine normalized an increased testosterone due to mSPS in male mice (Figure 2H). Pramipexole elevated testosterone in non-stressed males [*t*(36) = 2.609; *p* < 0.05] and had no effect in females (Figure 2G). Tiagabine increased female testosterone in the sham [*t*(37) = 3.003; *p* < 0.01] and mSPS-conditions [*t*(37) = 2.524; *p* < 0.05; Figure 2G].

Altogether, mSPS increased plasma testosterone in both sexes, and tiagabine increased testosterone in stressed and sham females, while pramipexole did so only in sham-stressed males.

### Associations Between Conditioned Freezing Response and Plasma Steroids

#### An Association Between Freezing Response and Plasma Corticosterone Levels

**FIGURE 3:**
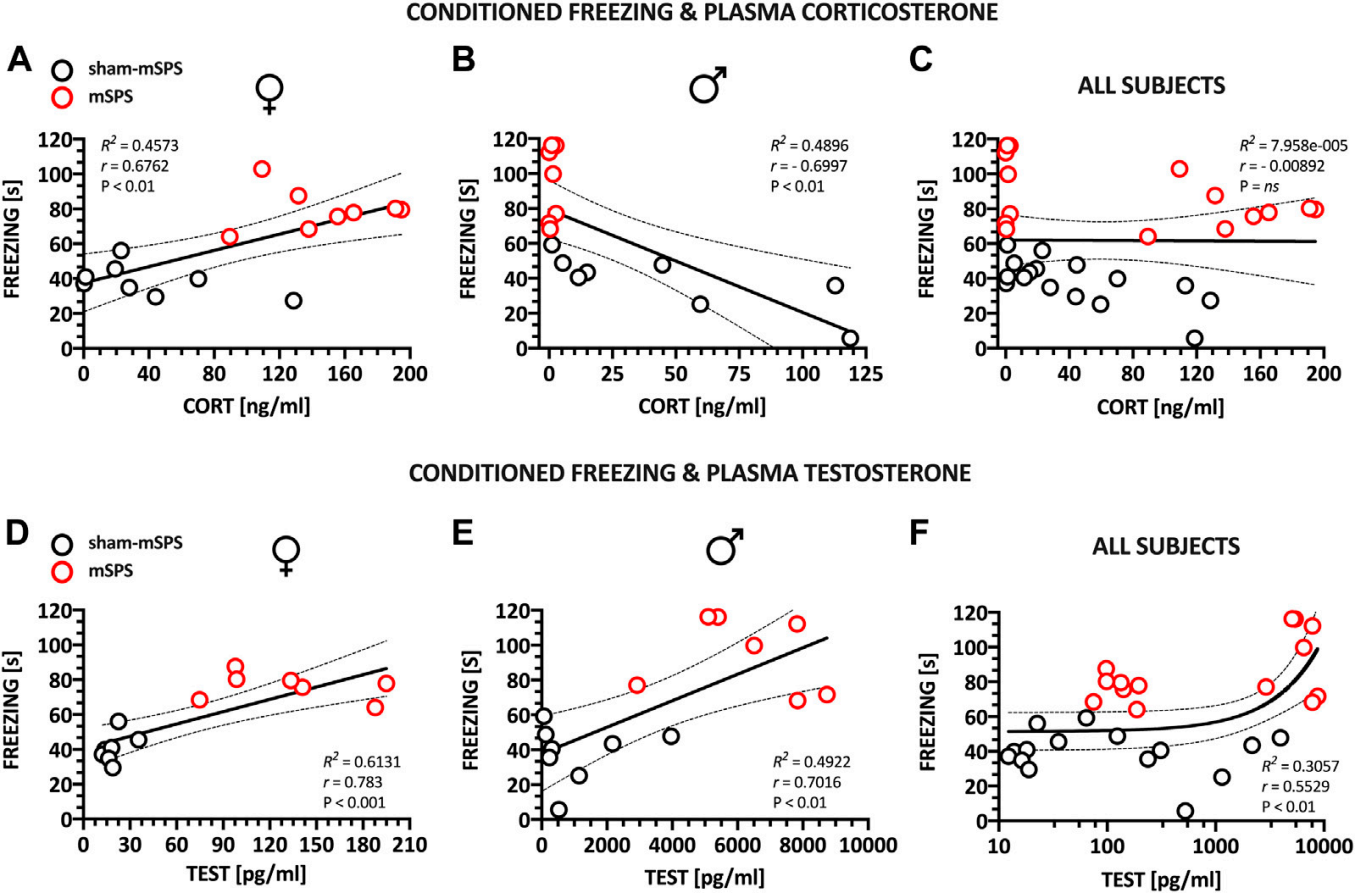
Correlations between behavioral and hormonal responses to trauma in female and male drug-naive mice, two weeks following mSPS. The duration of conditioned freezing was positively and negatively correlated with the plasma levels of corticosterone in female **(A)** and male mice **(B)**, respectively. No significant correlation was observed if the sex factor was collapsed **(C)**. Conditioned freezing was positively correlated with plasma testosterone in mice of both sexes (D**–**F). Regression analyses were used to determine *R* ^*2*^ and statistical significance. Correlated data sets were already presented as sham and mSPS controls treated with saline in the source Figures 2A,B for conditioned freezing, Figures 2C,D for plasma corticosterone and Figures 2G,H for plasma testosterone. For 17β-estradiol data, see Supplementary Materials. Abbreviations: 17β-ESTR–17β-estradiol, CORT—corticosterone, mSPS–mouse single prolonged stress, TEST–testosterone.

Figure 3A demonstrates the positive (*r* = =0.67; *R* ^*2*^ = =0.45; F[1,14] = =11.8; *p* P< 0.01) correlation between freezing response and plasma corticosterone levels in female mice. Figure 3B demonstrates negative [*r* = −0.69; *R* ^*2*^ = 0.48, F(1,13) = 12.47; *p* < 0.01] correlation between freezing response and plasma corticosterone levels in male mice. A similar analysis disregarding the sex of mice demonstrated no correlation between freezing and corticosterone levels (Figure 3C).

#### An Association Between Freezing Response and Plasma Estradiol Levels

There was no significant correlation between freezing response and plasma estradiol levels in female and male mice. For data and statistical analysis, see Supplementary Material.

#### An Association Between Freezing Response and Plasma Testosterone Levels

Figures 3D,E demonstrate significant positive correlation between freezing response and plasma testosterone levels in female [*r* = 0.78; *R* ^*2*^ = 0.61; F(1,12) = 19.02; *p* < 0.001] and male [*r* = 0.7; *R* ^*2*^ = 0.4922; F(1,13) = 2.92; *p* < 0.01] mice. A similar analysis disregarding the sex of mice also demonstrated positive correlation between freezing and testosterone levels [Figure 3F, *r* = 0.55; *R* ^*2*^ = 0.3057; F(1,27) = 11.89; *p* < 0.01].

## Discussion

This study examined whether sex affected behavioral and hormonal responses in a mouse model of PTSD-like symptoms and whether treatment with tiagabine or pramipexole could affect stress-induced phenotypes. While both females and males showed increased conditioned fear due to the trauma, hormonal responses were far more complex.

### Severe Stress Affects Behavior and Hormone Levels in Mice

#### Sex Differences in the Behavioral Response to Trauma

Earlier research, done chiefly in male rodents, demonstrate prolonged conditioned freezing several days following SPS (e.g., Keller et al., 2015; Malikowska-Racia et al., 2019a; for review, see; Verbitsky et al., 2020) and well correspond with the exaggerated fear response in PTSD patients as defined by DSM-5 (American Psychiatric Association, 2013). The twice higher prevalence of PTSD in women (Kessler et al., 1995; Yehuda et al., 2015) has been addressed in the SPS rat, but not mice studies. In this respect, traumatized female rats display more avoidance (Nahvi et al., 2019) and anhedonia-like behavior as well as social interaction impairments (Pooley et al., 2018), suggesting a stronger depressive-like phenotype. However, traumatized rat females are less than males prone to develop exaggerated conditioned fear (Keller et al., 2015). This corresponds with the Clark et al. (2019) findings from mice showing that also non-traumatized females manifest less robust though more persistent conditioned freezing than males.

Our mice’s results appear not to support the latter observations because we report prolonged conditioned freezing in both sexes. This discrepancy could be due to the species differences in response to trauma, the fact that we used isoflurane rather than ether anesthesia, some technical differences in the conditioned fear protocol, and/or other not identified factors. Nevertheless, from the fact that we observed increased conditioned freezing in both sexes (Figures 2A,B), one may conclude that the present experimental conditions fulfilled construct and face validities of mSPS as a PTSD model. Clearly, more research is needed to determine the optimal conditions for post-SPS/mSPS testing to reflect sex-dependent PTSD susceptibility in humans accurately.

#### Hormonal Sex Differences

##### Sex-dependent Effect on Plasma Corticosterone Level

Exposure to mSPS increased plasma corticosterone in females while reduced it in males (Figures 2A,B). Because both sexes showed trauma-induced prolonged conditioned freezing, corticosterone plasma levels were positively correlated with freezing duration in females and negatively correlated in males.

The release of cortisol in PTSD patients is abnormally reduced by HPA fast negative feedback (Yehuda et al., 1993; Yehuda et al., 2015), resulting in low cortisol that frequently correlates with the PTSD symptoms exacerbation (Aardal-Eriksson et al., 2001). Present report and other preclinical data on male mice agree with human findings (Liberzon et al., 1997; Kohda et al., 2007; Perrine et al., 2016; Pooley et al., 2018). The negative correlation between plasma corticosterone levels and conditioned freezing (Dopfel et al., 2019), extinction deficits, and other manifestations of PTSD-like behaviors (Danan et al., 2018) have already been reported in traumatized male rats.

Nevertheless, the simple causality of low cortisol and PTSD symptoms has already been challenged, both in rodent studies (e.g., Sillivan et al., 2017; Torrisi et al., 2021) and in the clinic. Reports on unchanged or even elevated cortisol in PTSD patients had prompted further research suggesting that low cortisol is associated with trauma exposure or symptoms severity (Aardal-Eriksson et al., 2001; Morris et al., 2016; Josephs et al., 2017; Speer et al., 2019). Beyond experimental heterogeneity that contributes to the contradictory reports (Speer et al., 2019), earlier research noted that lower cortisol had been more consistently reported in male than female subjects (Gill et al., 2005; Metzger et al., 2008). It agrees with our findings (Figures 3A–C) and suggests that studies disrespecting sex variable may bias final conclusions.

The pivotal role of sex in hormonal response to trauma is supported by an earlier rodent study by Pooley et al. (2018). These authors observed enhanced HPA control and low plasma corticosterone in traumatized male rats as well as reduced HPA negative feedback and higher corticosterone in females. The enhanced corticosterone response of traumatized female rodents resembles HPA hyposensitivity in depressive disorder and a “female”-specific PTSD-like phenotype (Pooley et al., 2018). While PTSD-suffering and depressed males display opposite HPA regulation manifesting as enhanced and decreased circulating glucocorticoids, females present HPA hyposensitivity and thus increased glucocorticoids in depression and PTSD (Yehuda, 2002; Kessler, 2003).

##### Effects on Plasma Testosterone and 17β-Estradiol

While stress exposure did not affect estradiol levels, testosterone increased in both sexes following trauma and its levels were positively correlated with the conditioned freezing. This agrees with Karlović et al. (2012), who showed increased testosterone levels in soldiers suffering PTSD. Testosterone’s protective role on the pathogenic effect of low cortisol has already been hypothesized (Josephs et al., 2017) and is supported by rodent results demonstrating that exogenous testosterone reduces PTSD-like symptoms (Fenchel et al., 2015; Lynch et al., 2016).

In contrast to testosterone, plasma levels of estradiol were not affected by trauma. It remains to be established whether estradiol plays a role in PTSD symptomatology or whether the progesterone/estradiol ratio may be essential for understating sex-dependent differences in fear and PTSD (Seligowski et al., 2020).

### Treatment Efficacy in Traumatized Male and Female Mice

The present study’s final goal was to examine whether treatment efficacy in a mouse PTSD-like model depended on the subjects’ sex. Stress-induced freezing was reduced by pramipexole in both sexes, but tiagabine decreased it only in female mice.

#### Sex-independent Efficacy of Pramipexole

The clinical efficacy of pramipexole in PTSD has not yet been established (https://clinicaltrials.gov, ref. no NCT03765138, Research Foundation for Mental Hygiene,I, 2019). Figure 2 demonstrates that pramipexole reduced exaggerated freezing in traumatized female and male mice, suggesting its potential efficacy in PTSD treatment.

The rationale of using dopamine-mimetics in PTSD treatment is based on two hypotheses, implying faster fear memory extinction (Gerlicher et al., 2018) and facilitated stress adaptation (Cabib and Puglisi-Allegra, 2012; Douma and de Kloet, 2020). PTSD patients showed reduced cerebrospinal dopamine metabolites (Geracioti et al., 2013) and increased baseline dopamine beta-hydroxylase (Hamner and Gold, 1998), suggesting that dopamine-driven mechanisms are impaired after trauma.

Present study showing that D_2_-like type receptors agonist reduced conditioned fear but not hormonal response suggests that pramipexole did not affect stress adaptation, supporting inefficacy of quinpirole to reduce plasma corticosterone in rats subjected to electric shock stressor (De Oliveira et al., 2013) or selegiline in mice exposed to forced swim (Kasai et al., 2017).

Of note, pramipexole increased testosterone levels in non-stressed males. It agrees with findings by Okun et al. (2014), which shows elevated testosterone due to pramipexole in men and corresponds with the postulated testosterone's protective role in low corticosterone conditions (Josephs et al., 2017).

#### Sex-dependent Efficacy of Tiagabine

Tiagabine has already been tested in PTSD patients, and while studies reported ambiguous results (Connor et al., 2006; Davidson et al., 2007), the medication presented greater efficacy in women, agreeing with present data (Figure 2A).

PTSD patients demonstrate deficits related to GABA-ergic neurotransmission (Geuze et al., 2008; Trousselard et al., 2016; Rasmusson et al., 2019), although, in women, they are additionally magnified by specific blocks in progesterone metabolism resulting in an insufficient GABA-ergic neurosteroid modulation (Pineles et al., 2018; Rasmusson et al., 2019). Thus, the sex-specific efficacy of tiagabine may rely on complementing GABA-ergic neurotransmission, which is impaired in women.

### Study Limitations

This study suffers from some limitations, including the use of isoflurane instead of diethyl ether as one of the mSPS components, which might have affected trauma efficacy (Knox et al., 2012). The use of isoflurane was due to the present ethical guidelines, but care was taken to anesthetize all mice efficiently. Second, between mSPS and the measurement of its effects, we repeatedly injected animals, though it is unlikely that the injections induced stress comparable with restraint or forced swim as in the rat study (Liberzon et al., 1999). Finally, in the present fear conditioning test, a solely contextual conditioned cue was applied as in the PTSD studies investigating trauma-unrelated conditioned fear. This approach has already been used to examine PTSD-like phenotype of SPS-exposed rats (e.g., Takahashi et al., 2006; Kohda et al., 2007) and is supported by Orr et al. (2000) findings showing that following *de novo* conditioning, patients demonstrated increased fear evoked by conditional visual cues.

## Conclusion

We report that plasma corticosterone depends on trauma and sex in mice suggesting sex-dependent HPA adaptation to severe stress purportedly biasing treatment outcome. Current results indicate the potential efficacy of D_2_-like receptor agonists in the treatment of PTSD in male and female subjects and of GABA-mimetics in females.

## Data Availability

The original contributions presented in the study are included in the article/Supplementary Material, further inquiries can be directed to the corresponding author.
