# Supplementary material for: Sex, Pramipexole and Tiagabine Affect Behavioral and Hormonal Response to Traumatic Stress in a Mouse Model of PTSD
Source: Front Pharmacol. 2021 Jun 30;12:691598. doi: 10.3389/fphar.2021.691598 (PMC8277945; doi:10.3389/fphar.2021.691598)
Supplement: Supplementary file 1 [file DataSheet1.docx]

Supplementary Material

# An association between freezing response and plasma 17β-estradiol levels


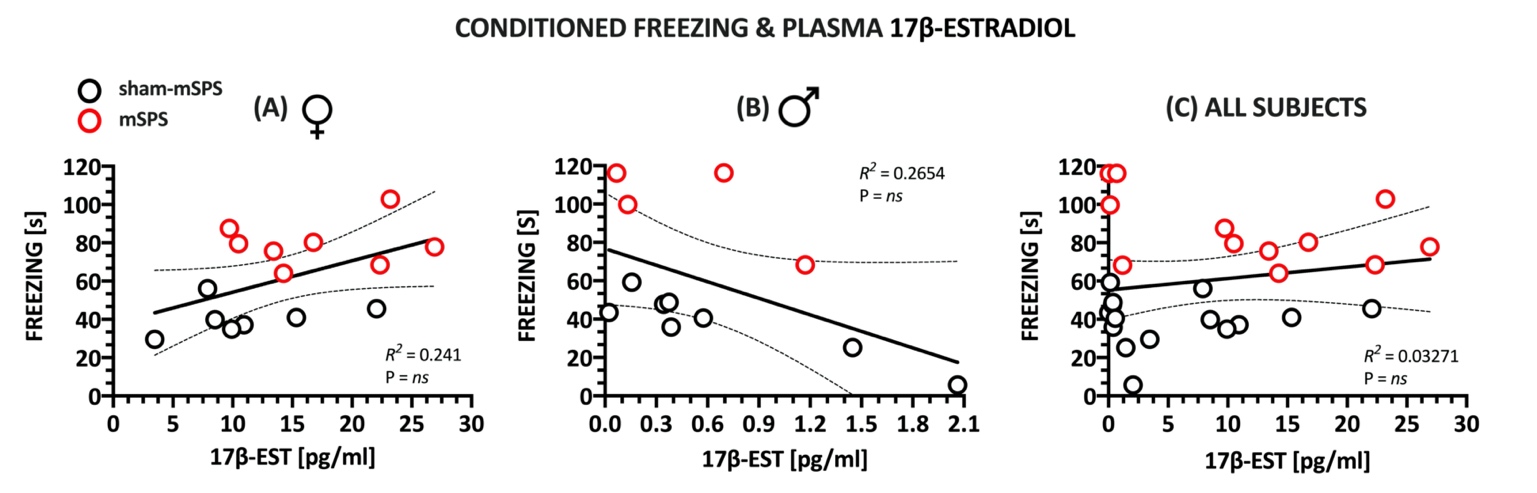
There was no significant correlation between freezing response and plasma estradiol levels in female and male mice or if sex was collapsed.

**Supplementary Figure 1. Conditioned freezing did not correlate with plasma estradiol in female mice (A), male mice (B) or if sex factor was collapsed (C).** Regression analyses were used to determine *R^2^* and statistical significance. The detailed statistic is included in the table below. Abbreviations: 17β-ESTR – 17β-estradiol, mSPS – mouse single prolonged stress.

# The detailed results of statistical analyses included in the article and supplementary materials.

**Table 1. ANOVA statistics**

| **Figure** | **Statistical method** | **Dependent variable** | **Effects of independent variables and their interactions** | | | | | | |
| --- | --- | --- | --- | --- | --- | --- | --- | --- | --- |
|  |  |  | **mSPS** | **Sex** | **Treatment** | **mSPS x Sex** | **mSPS x Treatment** | **Sex x Treatment** | **mSPS x Sex x Treatment** |
| **Fig 2A, 2B** | 3-way ANOVA^[[1]](#footnote-1)^a | Freezing time  [s] | *F[1,84] = 60.68*  ***P < 0.001***  partial η^2^ =0.419 | *F[1,84] = 2.6*  *NS*  partial η^2^ = 0.03 | *F[2,84] = 14.89*  ***P < 0.001***  partial η^2^ = 0.262 | *F[1,84] = 3.41*  ***P=0.068***  partial η^2^ = 0.039 | *F[2,84] = 5.35*  ***P < 0.01***  partial η^2^ = 0.113 | *F[2,84] = 0.297*  *NS*  partial η^2^ = 0.007 | *F[2,84] = 0.706*  *NS*  partial η^2^ = 0.017 |
| **Fig 2A** | 2-way ANOVA | Freezing time  [s] | *F[1,42] = 38.18*  ***P < 0.001***  partial η^2^ = 0.449 | *NA* | *F[2,42] = 16.41*  ***P < 0.001***  partial η^2^ = 0.439 | *NA* | *F[2,42] = 4.78*  ***P < 0.05***  partial η^2^ = 0.186 | *NA* | *NA* |
| **Fig 2B** | 2-way ANOVA | Freezing time  [s] | *F[1,42] = 31.309*  ***P < 0.001***  partial η^2^ = 0.427 | *NA* | *F[2,41] = 4.526*  ***P < 0.05***  partial η^2^ = 0.177 | *NA* | *F[2,41] = 2.418*  *NS*  partial η^2^ = 0.103 | *NA* | *NA* |
| **Fig 2C**  **Fig 2D** | 3-way ANOVA | CORT  [ng/ml] | *F[1,78] = 9.33*  ***P < 0.01***  partial η^2^ = 0.107 | *F[1,78] = 154*  ***P < 0.001***  partial η^2^ = 0.664 | *F[2,78] = 4.5*  ***P < 0.05***  partial η^2^ = 0.104 | *F[1,78] = 25.96*  ***P < 0.001***  partial η^2^ = 0.25 | *F[2,78] = 1.96*  *NS*  partial η^2^ = 0.048 | *F[2,78] = 10.34*  ***P < 0.001***  partial η^2^ = 0.21 | *F[2,78] = 10.03*  ***P < 0.001***  partial η^2^ = 0.204 |
| **Fig 2E**  **Fig 2F** | 3-way ANOVA^[[2]](#footnote-2)^a | 17β-ESTR  [pg/ml] | *F[1,71] = 7.3*  ***P < 0.01***  partial η^2^ = 0.093 | *F[1,71] = 46.02*  ***P < 0.001***  partial η^2^ = 0.395 | *F[2,71] = 4.06*  ***P < 0.05***  partial η^2^ = 0.103 | *F[1,71] = 0.46*  *NS*  partial η^2^ = 0.006 | *F[2,71] = 9.5*  ***P < 0.001***  partial η^2^ = 0.211 | *F[2,71] = 2.94*  *NS*  partial η^2^ = 0.076 | *F[2,78] = 0.8*  *NS*  partial η^2^ = 0.022 |
| **Fig 2E** | 2-way ANOVA | 17β-ESTR  [pg/ml] | *F[1,39] = 1.48*  *NS*  partial η^2^ = 0.037 | *NA* | *F[1,39] = 2.86*  *ns*  partial η^2^ = 0.126 | *NA* | *F[2,39] = 5.54*  ***P < 0.05***  partial η^2^ = 0.221 | *NA* | *NA* |
| **Fig 2F** | 2-way ANOVA | 17β-ESTR  [pg/ml] | *F[1,32] = 17.5*  ***P < 0.001***  partial η^2^ = 0.353 | *NA* | *F[1,32] = 9.87*  ***P < 0.001***  partial η^2^ = 0.382 | *NA* | *F[2,32] = 7.67*  ***P < 0.01***  partial η^2^ = 0.324 | *NA* | *NA* |
| **Fig 2G** | 2-way ANOVA | TEST  [pg/ml] | *F[1,37] = 13.2*  ***P < 0.001***  partial η^2^ = 0.263 | *NA* | *F[2,37] = 13.14*  ***P < 0.001***  partial η^2^ = 0.415 | *NA* | *F[2,37] = 0.75*  *NS*  partial η^2^ = 0.039 | *NA* | *NA* |
| **Fig 2H** | 2-way ANOVA | TEST  [pg/ml] | *F[1,37] = 36.3*  ***P < 0.0001***  partial η^2^ = 0.502 | *NA* | *F[2,36] = 3.82*  ***P < 0.05***  partial η^2^ = 0.175 | *NA* | *F[2,36] = 1.68*  *NS*  partial η^2^ = 0.085 | *NA* | *NA* |

**Table 2. Regression and correlation analyses.**

| **Correlation and regression analyses^[[3]](#footnote-3)^** | |
| --- | --- |
| **Fig 3A** | *R^2^*=0.45; F[1,14]=11.8; P<0.01; Adjusted *R^2^* = 0.4185; line: Y = 0.233*X + 37.52; slope = 0.233 ± 0.06783; 95%CI: 0.08749 to 0.3784; Y Intercept = 37.52 ± 7.724; 95%CI:20.95 to 54.09; *r*= 0.6762; 95%CI: 0.2716 to 0.8777 |
| **Fig 3B** | *R^2^*=0.48, F[1,13]=12.47; P<0.01; Adjusted *R^2^*= 0.4503; line: Y = -0.5881*X + 79.32; slope = -0.5881 ± 0.1666; 95%CI: -0.9479 to -0.2283; Y Intercept = 79.32 ± 7.794; 95%CI:62.48 to 96.16; *r* = -0.6997; 95%CI: -0.8922 to -0.2921. |
| **Fig 3C** | *R^2^*=7.958e-005, F[1,29]=2.308e-003; P=NS; P<0.01 Adjusted *R^2^* = -0.0344; line: Y = -0.003934*X + 62.02; slope = -0.003934 ± 0.0819; 95%CI: -0.1714 to 0.1636; Y Intercept = 62.02 ± 7.211; 95%CI: 47.27 to 76.76; *r* = -0,00892; 95%CI: -0,3621 to 0,3465 |
| **Fig 3D** | *R^2^*=0.6131; F[1,12]=19.02; P < 0.001; Adjusted *R^2^* = 0.5809, line: Y = 0.2357*X + 40.43; slope = 0.2357 ± 0.05405; 95%CI: 0.1179 to 0.3535; Y Intercept = 40.43 ± 5.385; 95%CI: 28.69 to 52.16; *r*= 0.7830; 95%CI: 0.4318 to 0.928 |
| **Fig 3E** | *R^2^*=0.4922; F[1,13]=2.92; *P < 0.01*; Adjusted *R^2^* = 0.4532, line: Y = 0.007598*X + 37.71; slope = 0.007598 ± 0.00214; 95%C: 0.002974 to 0.0122; Y Intercept = 37.71 ± 9.994; 95%CI: 16.12 to 59.30; *r*= 0.7016; 95%CI: 0.2956 to 0.8929 |
| **Fig 3F** | *R^2^*=0.3057; F[1,27]=11.89; *P < 0.01*; Adjusted *R^2^* = 0.28, line: Y = 0.005446*X + 51.41; slope = 0.005446 ± 0.00214; 95%C: 0.002205 to 0.008687; Y Intercept = 51.41 ± 9.994; 95%CI: 40.52 to 62.3; *r*= 0.5529; 95%CI: 0.2338 to 0.7645 |
| **Suppl Fig 1A** | R^2^=0.241; F[1,13]=4.128; P = 0.063; Adjusted R^2^ = 0.1826, line: Y = 1.647*X + 37.70; slope = 1,647 ± 0,8105; 95%CI: -0.1043 to 3.398; YIntercept = 37.7 ± 12.76; 95%CI: 10.13 to 65.27; r= 0.49; 95%CI: -0.02852 to 0.8016 |
| **Suppl Fig 1B** | R^2^=0.2654; F[1,10]=3.614; P = 0.0865; Adjusted R^2^ = 0.1920, line: Y = Y = -28.73*X + 76.71; slope = -28.73 ± 25.11; 95%CI: -62.41 to 4.945; YIntercept = 76.71 ± 13.08; 95%CI: 47.55 to 105.9; r= - 0.5152; 95%CI: - 0.8406 to 0.0833 |
| **Suppl Fig 1C** | *R^2^=0.03271; F[1,25]=0.8453; P = 0.36; Adjusted R^2^ = -0.00598, line: Y = Y = 0.5991*X + 55.31; slope = 0.5991 ± 0.6516; 95%CI: -0.7429 to 1.941; YIntercept = 55.31 ± 7.657; 95%CI: 39.54 to 71.08; r= - 0.1808; 95%CI: -0.2139 to 0.5248* |

1. a Due to insignificant third-order interaction, but significant effects of treatment and mSPS factors and their interactions, analyses were split out into two 2-way ANOVAs based on sex.

   Abbreviations: 17β-ESTR – 17β-estradiol, ANOVA – analysis of variance, CORT – corticosterone, NA – not applicable, NS: P > 0.05, TEST –testosterone. [↑](#footnote-ref-1)
2. a Due to insignificant third-order interaction, but significant effects of treatment and mSPS factors and their interactions, analyses were split out into two 2-way ANOVAs based on sex.

   Abbreviations: 17β-ESTR – 17β-estradiol, ANOVA – analysis of variance, CORT – corticosterone, NA – not applicable, NS: P > 0.05, TEST –testosterone. [↑](#footnote-ref-2)
3. Abbreviations: 95% CI : 95% confidence interval, NS: P > 0.05. [↑](#footnote-ref-3)
